# Psychometric validation of a community healthcare scorecard used for marginalized populations in Bangladesh

**DOI:** 10.1371/journal.pone.0348720

**Published:** 2026-08-24

**Authors:** Md. Talha Jubaer, Md. Muhitul Alam, Md. Israt Rayhan, Nayeem Sultana, Abu Said Md Juel Miah, Mohd. Rubayat Ahsan

**Affiliations:** 1 Institute of Statistical Research and Training (ISRT), University of Dhaka, Dhaka, Bangladesh; 2 Department of Development Studies, University of Dhaka, Dhaka, Bangladesh; 3 Department of Sociology, Loyola University Chicago, Chicago, Illinois, United States of America; 4 Research and Policy, BRAC, Dhaka, Bangladesh; Federal University of Santa Maria: Universidade Federal de Santa Maria, BRAZIL

## Abstract

**Background:**

Achieving equitable and quality healthcare for marginalized groups remains a critical challenge in Bangladesh. Community perceptions are crucial for assessing healthcare system performance, yet tools like Community Scorecards (CSCs) often lack rigorous psychometric validation, limiting their usefulness for evidence-based policy decisions.

**Objective:**

This study aims to psychometrically validate a 14-item CSC for measuring perceived healthcare service quality among marginalized populations in Bangladesh, using both Classical Test Theory (CTT) and the Generalized Partial Credit Model (GPCM) from Item Response Theory.

**Methods:**

Data were collected in 2023 from 311 community scoring units across nine marginalized population groups. Each scoring unit represents a Focus Group Discussion (FGD) in which participants reached a group consensus rating on each item using the five-point Likert scale (1 = Very Bad to 5 = Very Good) provided in the questionnaire. We conducted exploratory analysis, factorability checks including the Kaiser-Meyer-Olkin measure and Bartlett’s test of sphericity, exploratory factor analysis (EFA) with oblique (promax) rotation, reliability assessment using both Cronbach’s alpha and McDonald’s omega, and GPCM analysis to evaluate item and scale performance.

**Results:**

The Kaiser-Meyer-Olkin measure confirms excellent sampling adequacy (0.83), and Bartlett’s test of sphericity was significant (χ² = 1392.90, df = 91, p < 0.001), supporting factorability. EFA reveals a three-factor structure (Accessibility and Fairness, Institutional Responsiveness, Maternal and Child Health Support) explaining 52.7% of variance. The scale shows high internal consistency (Cronbach’s α = 0.84; McDonald’s ω = 0.87). Although three correlated factors emerge at the CTT level, the GPCM is estimated under a unidimensional assumption empirically supported by a dominant first factor (eigenvalue 4.64 versus 1.58 for the second) and by a moderate average inter-item correlation (0.27). On model fit, the GPCM was preferred over a more restrictive partial credit model on both AIC and BIC, and no item showed systematic residual misfit. GPCM results indicate that all items have significant, positive discrimination parameters (range 0.47 to 2.50), with fairness and access items being the most discriminating. Although the response options were originally designed as a five-point scale, category 5 (Very Good) was not endorsed for any health item, so the GPCM was effectively estimated on the four empirically used categories. Category thresholds were generally well ordered and most transitions were statistically significant, though a small number of intermediate thresholds were not, suggesting that some adjacent categories may be less clearly separated in respondents’ minds and warrant attention in future revisions. The Test Information Function shows peak measurement precision for respondents with low to moderate perceived quality levels.

**Conclusion:**

The CSC appears to be a psychometrically sound and contextually relevant instrument for the populations studied. Its precision in measuring lower quality perceptions makes it particularly useful for monitoring disparities and evaluating interventions targeting underserved populations. Confirmatory testing in independent samples and additional checks of local independence will further strengthen confidence in its dimensional structure.

## Introduction

Achieving equitable and quality healthcare remains a formidable challenge in low- and middle-income countries (LMICs), despite its centrality to the Sustainable Development Goals [1]. While global efforts have expanded the coverage of essential services, deficits persist in the effectiveness, safety, and responsiveness of health systems that are fundamental to good health outcomes [1,2]. These shortcomings fall disproportionately on marginalized populations, who experience intersecting barriers of poverty, adverse geographic locations, gender disparity, disability, and social stigma and exclusion [3]. For these groups, access to healthcare is impeded not only by a lack of facilities but also by prohibitive costs, long waiting times, discriminatory provider attitudes, and a profound absence of accountability, all of which shape their perceptions of healthcare quality [4].

Over the past two decades, the case for measuring and integrating community and patient perspectives into health system evaluation has drawn significant attention from policy actors and researchers [5,6]. Community perceptions of service quality help determine healthcare-seeking behavior, treatment adherence, and trust in the system, which makes them indispensable for people-centered policy and system reforms [5,7,8]. Traditional monitoring frameworks rely heavily on administrative data and clinical indicators, and they often miss the real-life experiences of marginalized communities [9,10]. To address this gap, participatory social accountability tools such as the Community Scorecard (CSC) have been developed and used to generate evidence-based grassroots feedback on service delivery [11,12]. In Bangladesh, the use of such tools is increasingly taken into action to identify service gaps and amplify community voices [11,13]. Nonetheless, a critical limitation of these tools persists: the psychometric properties of these instruments are seldom rigorously established, which undermines their credibility and policy-level usefulness [14,15].

Validating measurement tools is a scientific prerequisite. Classical Test Theory (CTT) offers foundational techniques, including exploratory factor analysis and reliability assessment, to evaluate a scale’s internal structure and consistency [14,16]. CTT is limited, however, by its sample dependence and by the assumption that all items contribute equally to measuring a latent trait [17]. Item Response Theory (IRT), and in particular the Generalized Partial Credit Model (GPCM) for polytomous responses, provides a more granular framework: it estimates item-specific discrimination parameters and threshold parameters for each response category transition, allowing researchers to evaluate whether the scale is sensitive across the entire continuum of the trait [18–20].

In Bangladesh, despite notable progress in health indicators, stark inequities in healthcare access and quality persist [13,21], and the validation of community monitoring tools overwhelmingly relies on CTT [11,13]. This gap is consequential, as marginalized populations often exhibit complex response patterns shaped by varied experiences of exclusion, which require careful modeling for accurate interpretation [17].

The 14 items used in this study, developed through a thorough exercise on sub-indicators of quality and equitable health services, were not newly developed but were drawn from an existing CSC instrument developed and implemented by BRAC Bangladesh. The original item pool was built on three sources: international scholarship on healthcare quality and patient experience [5,6,12,22,23], domains commonly tracked in Bangladeshi health system reviews [13,21], and qualitative scoping work with marginalized communities that surfaced locally salient concerns about access, fairness, accountability, and maternal and child services. The questionnaire was prepared in English, translated into Bangla, and then back-translated by an independent bilingual researcher; discrepancies were resolved by consensus. An expert panel of public health researchers, BRAC program staff, and community workers reviewed the items for content relevance, cultural appropriateness, and clarity of wording. A pre-test with two community groups was conducted before full data collection to check whether the Likert categories and statement phrasings were easy to comprehend; a rigorous revision of question structure and response options followed the pre-test, intending to reduce interviewer and response errors. These steps were intended to support content validity, although a structured content validity index was not computed and is acknowledged as a limitation.

This study aims to provide a comprehensive psychometric evaluation of the 14 items used in a Community Scorecard with various marginalized populations in Bangladesh. Using an integrated CTT and IRT strategy, our objectives are to identify the dimensional structure of community-defined quality healthcare, to establish reliability and internal consistency, and to use the GPCM for an item- and category-level evaluation of discrimination, threshold ordering, and measurement precision. By offering a rigorously evaluated, context-sensitive tool, this work seeks to support evidence-based accountability and equity-focused policy interventions in Bangladesh and similar settings [1,11,24].

## Materials and methods

### Data and sampling

Data were collected in 2023 through a Community Scorecard (CSC) exercise designed by BRAC and implemented by the Centre for Research and Development (CRD), Dhaka. The CSC is a participatory monitoring tool that combines quantitative ratings on a five-point Likert scale (Very Bad = 1 to Very Good = 5) with qualitative insights from facilitated group discussions, and it has been applied across urban, rural, hard-to-reach, disaster-prone, and institutional settings to capture the perspectives of populations typically underrepresented in conventional surveys.

The sampling structure proceeded in clearly defined steps. First, all 64 districts of Bangladesh were included in the sampling frame. Within each district, a list of communities representing the nine eligible marginalized groups (lower-caste Hindus known as Dalits, ethnic minorities or indigenous peoples, third gender individuals known as hijras, persons with disabilities, internally displaced people, Bede or river gypsies, elderly persons involved in begging, the urban floating population, and people living in geographically hard-to-reach areas such as river islands (chars), wetland areas (haors), hills, and islands in the sea) was compiled with the help of BRAC field offices and local civil society partners. From this list, five marginalized communities per district were randomly selected. Within each selected community, one Focus Group Discussion (FGD) was conducted with 8–12 purposively recruited adult participants who reflected diversity in age, gender, occupation, and length of residence; the FGDs, facilitated by trained enumerators, yielded a single agreed score per item for that community. The target sample was therefore 64 districts × 5 communities = 320 community-level FGDs; nine units were excluded for missing item scores, yielding the analytic sample of 311 FGDs used throughout the analysis.

The unit of analysis is the FGD (community-level scoring unit), not the individual participant. Each row in the analytic dataset corresponds to one FGD, and each numerical rating reflects the consensus reached by the group after facilitated discussion. We use the term ‘respondent’ in this paper to refer to a scoring unit (an FGD) rather than to an individual. This design choice was deliberate: the Community Scorecard is intended to capture a community-level judgment about service quality rather than a strictly personal opinion, and consensus scoring is one of the defining methodological features of the CSC tradition [11,12].

This consensus structure has consequences for the independence assumption that underlies IRT estimation. Independence at the level of analysis (the FGD) is plausible because each consensus score was generated by a different group of participants in a different community, and the random selection of communities within districts reduces concerns about systematic dependence across units. Preliminary checks of intra-district correlations did not detect strong district-level clustering, and we did not include district random effects in the GPCM because each district contributed only a small number of FGDs. We acknowledge, however, that within-FGD individual opinions may be correlated and that vocal participants may shape group ratings; this is a recognized limitation of consensus-based scorecard methods and is reflected in the limitations section.

Before each FGD, enumerators introduced themselves and their institutional affiliation, explained the objectives of the study, described the CSC as a participatory tool, and explained the Likert scale rating system together with the open-ended discussion component. Informed and written consent was obtained from all respondents. Participants were given the opportunity to ask questions before consenting, and they were informed that participation was voluntary and that they could withdraw at any time without consequence. The study exclusively involved adults aged 18 years and above; no minors were interviewed. The primary measurements are the ordinal community-level ratings on each CSC item; these ratings serve as the dependent variables in the GPCM, which models the probability of selecting a given category as a function of a latent perceived quality trait and item-specific threshold parameters [25].

### Ethics

This study was carried out as part of a research program commissioned by BRAC Bangladesh and implemented by the Centre for Research and Development (CRD), Dhaka. At the time the fieldwork was undertaken, the implementing organization did not maintain a standing Institutional Review Board (IRB), and formal external IRB review was not available within the project timeline. However, a rigorous ethical review of protocols and oversight of data collection were exercised through internal research governance procedures developed by BRAC and CRD, which included senior researcher review of study protocols and data collection instruments, supervisor oversight of fieldwork, and adherence to BRAC’s institutional code of conduct for research with vulnerable populations. All enumerators received a three-day training on informed consent, confidentiality, respectful conduct with marginalized groups, and protocols for handling distress or sensitive disclosures during FGDs. Written informed consent was obtained from every participant, no personally identifying information was retained in the analytic dataset, and audio recordings (where consented to) were stored on password-protected devices. We recognize that the absence of formal external IRB approval is a limitation that affects the international transferability of the work.

### Statistical analysis

The statistical analysis examined the structure, reliability, and measurement performance of the 14-item healthcare service quality scale. Both Classical Test Theory (CTT) and Item Response Theory (IRT) approaches were applied to provide complementary evidence on construct validity, internal consistency, and item-level functioning. Analyses were conducted sequentially, beginning with exploratory data analysis, followed by factor analysis, reliability assessment, and IRT modeling.

### Exploratory data analysis

Preliminary screening assessed missing data patterns, response distributions, and potential outliers. Item-level descriptive statistics (means, standard deviations, minimums, maximums) were calculated to evaluate response variability and floor or ceiling effects. Frequency distributions were examined to ensure that all response categories were adequately used, and inter-item correlations were inspected to assess coherence among items and to identify relationships consistent with psychometric scale development [15].

### Assessment of factorability

The suitability of the item set for factor analysis was evaluated using the Kaiser-Meyer-Olkin (KMO) Measure of Sampling Adequacy. The KMO statistic assesses the extent to which observed correlations among items can be explained by underlying latent factors rather than random noise. Overall and item-level KMO values were examined, with values exceeding 0.60 interpreted as acceptable and values above 0.80 as good to excellent [26]. In addition, Bartlett’s test of sphericity was conducted to verify that the correlation matrix differed significantly from an identity matrix; a significant test result is a necessary condition for proceeding with factor extraction.

### Exploratory factor analysis

Exploratory factor analysis (EFA) was conducted to identify the latent dimensions underlying community perceptions of healthcare service quality. A common factor extraction approach was employed, focusing on shared variance among items, with initial communalities estimated by squared multiple correlations. Because the underlying factors representing different aspects of healthcare quality were expected to be correlated rather than independent, an oblique rotation (promax) was applied; this allowed factors to covary and yielded a more interpretable simple structure than orthogonal rotation. Factor retention followed multiple criteria considered jointly: the eigenvalue-greater-than-one rule, inspection of the scree plot, the proportion of variance explained, and the substantive interpretability of the resulting factors [27]. For item retention, a primary loading of 0.40 or greater on a target factor was treated as practically meaningful, and items with cross-loadings within 0.20 of the primary loading were flagged for inspection. Uniqueness values (the proportion of an item’s variance not accounted for by the extracted factors) were also examined; values exceeding approximately 0.70 were noted as indicating that the item carried a substantial amount of variance unrelated to the common factors, which may reflect either item-specific content or measurement error.

### Reliability analysis

Internal consistency reliability of the overall scale was evaluated using Cronbach’s alpha [16]. Item-total correlations and ‘alpha if item deleted’ values were examined to identify items that contributed weakly to the overall scale. Because Cronbach’s alpha assumes essential tau equivalence (equal factor loadings), which is rarely tenable in multidimensional scales, we additionally computed McDonald’s omega based on the standardized factor loadings and uniqueness; omega is generally regarded as a more appropriate index of reliability for scales with a non-parallel item structure. Subscale-level alpha and omega were also computed for each of the three factors retained from the EFA.

### Item response theory analysis: Generalized partial credit model

To evaluate item and response category functioning beyond CTT, the Generalized Partial Credit Model (GPCM) was applied [20]. The GPCM is an IRT model for polytomous, ordered response categories and allows item discrimination to vary across items, which is particularly useful for healthcare service quality scales where items may differ in their capacity to distinguish among respondents [18,28,29]. Because the GPCM as specified in this study assumes a single latent trait, we first verified that a unidimensional approximation was empirically defensible despite the three-factor CTT solution. Three pieces of evidence supported this approach: (a) the first eigenvalue (4.64) was substantially larger than the second (1.58) and third (1.16), indicating a dominant general factor; (b) all 14 items loaded positively and meaningfully on the first unrotated factor, with loadings ranging from 0.43 to 0.73; and (c) the moderate average inter-item correlation (0.27) is consistent with a measurable common construct underlying perceived service quality. We therefore interpret the GPCM as estimating a general perceived service quality trait, while acknowledging that the multidimensional structure documented in the EFA implies meaningful subdomains that warrant further modeling (for example, bifactor or multidimensional IRT) in larger samples.

Under the GPCM, a single latent trait θ representing perceived healthcare service quality was estimated for each scoring unit. For an item *i* with *m*_*i*_ response categories, the probability that a unit with trait θ selects category *k* is given by


$$\begin{array}{c} P\left(X_{i}=k|\theta \right)=\frac{exp\left(\sum_{h=\text{1}}^{k}a_{i}\left(\theta -b_{ih}\right)\right)}{\sum_{c=\text{0}}^{m_{i}}exp\left(\sum_{h=\text{1}}^{c}a_{i}\left(\theta -b_{ih}\right)\right)}, \end{array}$$ (1)

where *a*_*i*_ is the item discrimination parameter and *b*_*ih*_ are the threshold parameters for transitions between adjacent categories. Item discrimination parameters were examined to assess how well items differentiated respondents along the latent trait; threshold parameters were examined to assess the relative difficulty of moving between adjacent categories. The latent trait was assumed to follow a standard normal distribution for model identification.

Model fit was evaluated at both the global and item levels. Global fit was assessed using the log-likelihood at convergence, model convergence checks, and information criteria (AIC and BIC) compared against a more restrictive partial credit model in which discrimination was constrained to be equal across items; the GPCM was preferred on both AIC and BIC, supporting the use of item-specific discrimination parameters. Item-level fit was inspected through observed versus expected category response curves and through standardized residuals across trait deciles; no item showed systematic deviation large enough to warrant exclusion. The assumption of local independence was examined by inspecting residual inter-item correlations after partial out the estimated latent trait; the largest residual correlations (between the two grievance items, and between the two maternal and child health items) were modest, consistent with the multidimensional structure detected in the EFA, and we discuss their implications in the Discussion. Item characteristic curves were inspected to evaluate category ordering, item information curves to assess precision contributed by individual items, and the test information function to evaluate overall measurement precision.

## Results

All statistical analyses were conducted using Stata (version 17) and R (version 4.3.1).

### Exploratory data analysis

Table 1 presents the community-wise distribution of the 311 FGDs in the analytic sample. The largest proportion of scoring units came from Dalit communities (18.65%), followed by persons with disabilities (17.04%) and elderly people involved in begging (11.90%). Third gender individuals accounted for 10.29% of the sample, while Bede (river gypsies) and ethnic minorities represented 9.97% and 9.00%, respectively. Smaller but comparable shares were observed among poor people living in hard-to-reach areas, the urban floating population, and internally displaced populations, each comprising about 7–8%.

**Table 1:** Type of marginalized community across the analytic sample (N = 311 FGDs).

Type of Community	Frequency	Percent (%)
Bede (river gypsies)	31	9.97
Poor people living in hard-to-reach areas	24	7.72
Third gender (hijra)	32	10.29
Dalits	58	18.65
Persons with disabilities	53	17.04
Elderly people involved in begging	37	11.90
Ethnic minorities/indigenous peoples	28	9.00
Urban floating people	24	7.72
Internally displaced population	24	7.72
**Total**	**311**	**100.00**

Descriptive statistics for the 14 items are shown in Table 2. Items related to overall service availability and core facility functions received comparatively higher ratings: government healthcare services (M = 2.57), referral systems (M = 2.50), and child care services (M = 2.74) were among the most favorably rated. Items capturing institutional responsiveness and accountability received the lowest ratings: grievance mechanisms (M = 1.48) and complaint handling (M = 1.37) showed pronounced and consistent dissatisfaction. Items related to access, information dissemination, fairness, and basic service conditions fell in a mid-range, with means generally between 2.05 and 2.30. A noteworthy feature of the data is that no FGD endorsed the highest category (5 = Very Good) on any of the 14 health items; observed responses were therefore concentrated in the lower four categories (1–4). This means that although the questionnaire was designed with a five-point response scale, the GPCM was effectively estimated over four empirically used categories. This pattern is informative in its own right: it suggests that none of the 311 community groups perceived their local government health services as ‘very good’, which is consistent with the broader literature on dissatisfaction with public sector primary care in marginalized settings in Bangladesh [21,30].

**Table 2:** Descriptive statistics of healthcare service quality items (N = 311).

Item	Mean	SD	Min	Max
Availability of public healthcare	2.57	0.79	1	4
Access to public healthcare	2.35	0.64	1	4
Information services	2.24	0.63	1	4
Equal access	2.12	0.68	1	4
Non-discrimination	2.21	0.69	1	4
Referral system	2.50	0.72	1	4
Grievance mechanism	1.48	0.76	1	4
Complaint handling	1.37	0.67	1	3
Patient-doctor ratio	2.30	0.65	1	4
Number of in-patient beds	2.09	0.58	1	3
Safe drinking water	1.99	0.61	1	4
Breastfeeding support	2.05	0.85	1	4
Mother care	2.44	0.64	1	4
Child care	2.74	0.53	1	4

### Results from factorability tests

The overall KMO value for the 14-item scale was 0.83, exceeding the commonly recommended minimum threshold of 0.60 and indicating good to excellent sampling adequacy. Item-level KMO values ranged from 0.66 to 0.92, with the two grievance items showing the lowest values (0.66 to 0.67), still above the minimum acceptable threshold of 0.50. Bartlett’s test of sphericity yielded χ² = 1392.90 (df = 91, p < 0.001), strongly rejecting the null hypothesis that the correlation matrix is an identity matrix. Taken together, these two diagnostics confirm that the item set is factorable.

### Results from factor analysis

Three factors were retained based on eigenvalues greater than one, inspection of the scree plot, and substantive interpretability. The three retained factors have eigenvalues of 4.64, 1.58, and 1.16, jointly accounting for 52.7% of the total variance. The likelihood ratio test of independence versus the saturated model is highly significant (χ² (91) = 1350.47, p < 0.001), further supporting the presence of meaningful common factors.

With promax (oblique) rotation, Factor 1 is defined primarily by accessibility, fairness, and resource adequacy items (access to the public healthcare system, equal access, non-discrimination, adequate information, service quality, referral system); we label this factor Accessibility and Fairness. Factor 2 is clearly defined by the two grievance items (grievance mechanism and complaint handling) and represents Institutional Responsiveness. Factor 3 captures facility conditions and maternal and child health amenities (patient-doctor ratio, number of in-patient beds, safe drinking water, breastfeeding support, mother care, child care) and is labeled Maternal and Child Health Support. No item shows problematic cross-loadings exceeding the 0.20 threshold from its primary loading, although a small number of items (notably availability of public healthcare and referral system) have relatively modest primary loadings and high uniqueness (0.78 and 0.63 in the unrotated solution, 0.86 and 0.77 after rotation). High uniqueness suggests that these items carry a substantial amount of variance not shared with the rest of the scale; this may reflect either item-specific content (for example, referral systems are an institutional feature that is only partly captured by perceived service quality) or scope for refining the item wording in future revisions. They were retained because of their substantive meaningfulness, and their removal did not improve overall scale reliability.

### Results from reliability analysis

Internal consistency of the 14-item scale is high. Cronbach’s α for the full scale is 0.839. McDonald’s ω, which does not assume tau-equivalent loadings, is 0.87 for the full scale, slightly higher than alpha and consistent with the expectation that omega tends to be more accurate when factor loadings vary across items.

Item-test correlations are all positive (range 0.46 to 0.70), and item-rest correlations, ranging from 0.35 to 0.62, indicate that each item contributes meaningfully to the total score. The average inter-item correlation is 0.27, within the acceptable range for unidimensional scales. Removing any single item does not result in a substantive improvement in alpha. Subscale reliability was also examined. The Accessibility and Fairness factor shows strong reliability (α = 0.81; ω = 0.86). The Institutional Responsiveness factor, although composed of only two items, displays strong internal consistency (α = 0.86; ω = 0.94). The Maternal and Child Health Support factor shows moderate reliability (α = 0.56; ω = 0.76), reflecting its smaller size and somewhat broader content; this lower subscale alpha is a reminder that the multidimensional structure is real and that interpretation of this subscale in isolation need be cautious.

### Results from the generalized partial credit model

The GPCM converges successfully (N = 311; log-likelihood = −3729.07). All 14 items have statistically significant and positive discrimination parameters (range 0.47 to 2.50, all p < 0.001), indicating that each item discriminates between FGDs with lower and higher levels of perceived service quality. Fairness, equal access, and information items show the highest discrimination, while breastfeeding support and mother and child healthcare show the lowest. On model fit, the GPCM outperforms a more restrictive partial credit model (in which discrimination is constrained to be equal across items) on both AIC and BIC, supporting the use of item-specific discrimination parameters. Inspection of standardized residuals across trait deciles does not identify any item with systematic misfit large enough to warrant removal. The largest residual inter-item correlations after partial out θ are between the two grievance items and between the two maternal items; these residuals are modest in magnitude but consistent with the multidimensional structure documented in the EFA.

### Item thresholds and overall scale performance

Threshold estimates from the GPCM are generally well ordered. For most items the first threshold (Category 2 vs. 1) is negative and statistically significant, suggesting that FGDs readily moved away from the lowest rating with relatively small improvements in perceived quality. The second thresholds (Category 3 vs. 2) show moderate difficulty and are statistically significant for key domains including accessibility, information provision, fairness, and service delivery. Third thresholds (Category 4 vs. 3) are consistently large and highly significant, implying that a rating of ‘Good’ reflects distinctly positive and comparatively rare service experiences, particularly in relation to institutional mechanisms and maternal and child healthcare services. A more cautious reading is warranted for a small number of intermediate thresholds. The second threshold (3 vs. 2) is not statistically significant for the availability of public healthcare item (p = 0.135), the referral system item (p = 0.105), and the mother care item (p = 0.321), and the first threshold (2 vs. 1) is not significant for the breastfeeding support item (p = 0.134). In a strict sense, a non-significant threshold means that the data do not place this transition point with precision, which suggests that the two adjacent categories may not be clearly separated in respondents’ minds for those items. Two non-mutually exclusive explanations are plausible: first, very few FGDs occupied those intermediate categories for these items (so the model has less information to estimate the threshold), and second, the wording of these items may invite less differentiation between ‘Bad’ and ‘Fair’ responses than items framed around discrimination or grievance. We therefore characterize the response category structure as substantially but not uniformly well functioning; the bulk of evidence supports the polytomous scale, but the items flagged above are candidates for refinement, possibly through cognitive interviewing or rewording, before the next round of data collection (Table 3).

**Table 3:** Generalized partial credit model estimates for healthcare service quality items (N = 311).

Item	Discrim.	*b*_*1*_ (2 vs 1)	*b*_*2*_ (3 vs 2)	*b*_*3*_ (4 vs 3)
Availability of public healthcare	0.62	−2.96***	−0.34	2.84***
Access to public healthcare	1.64	−1.95***	0.26*	3.38***
Information services	1.71	−1.69***	0.56***	3.94***
Equal access	2.50	−1.11***	0.71***	2.63***
Non-discrimination	2.38	−1.27***	0.53***	2.57***
Referral system	1.09	−2.15***	−0.23	2.85***
Grievance mechanism	0.81	1.65***	1.01***	4.35***
Complaint handling	1.08	1.56***	1.17***	–
Patient-doctor ratio	1.24	−1.99***	0.42***	4.26***
Number of in-patient beds	1.00	−2.09***	1.42***	–
Safe drinking water	0.69	−2.12***	2.23***	6.62***
Breastfeeding support	0.47	−0.46	0.44	5.84***
Mother care	0.98	−2.42***	−0.15	5.20***
Child care	1.03	−3.06***	−1.36***	4.54***

Note: *** p < 0.001; * p < 0.05. Discrimination parameters are item specific (a_i_). Thresholds (b_ih_) are estimated transitions between adjacent ordered response categories. A dash for the b_3_ entries for complaint handling and number of in-patient beds indicates that the highest response category (4) was not observed for those items, so the corresponding threshold is not estimable.

### Category Characteristic Curves (CCC)

**Fig 1:**
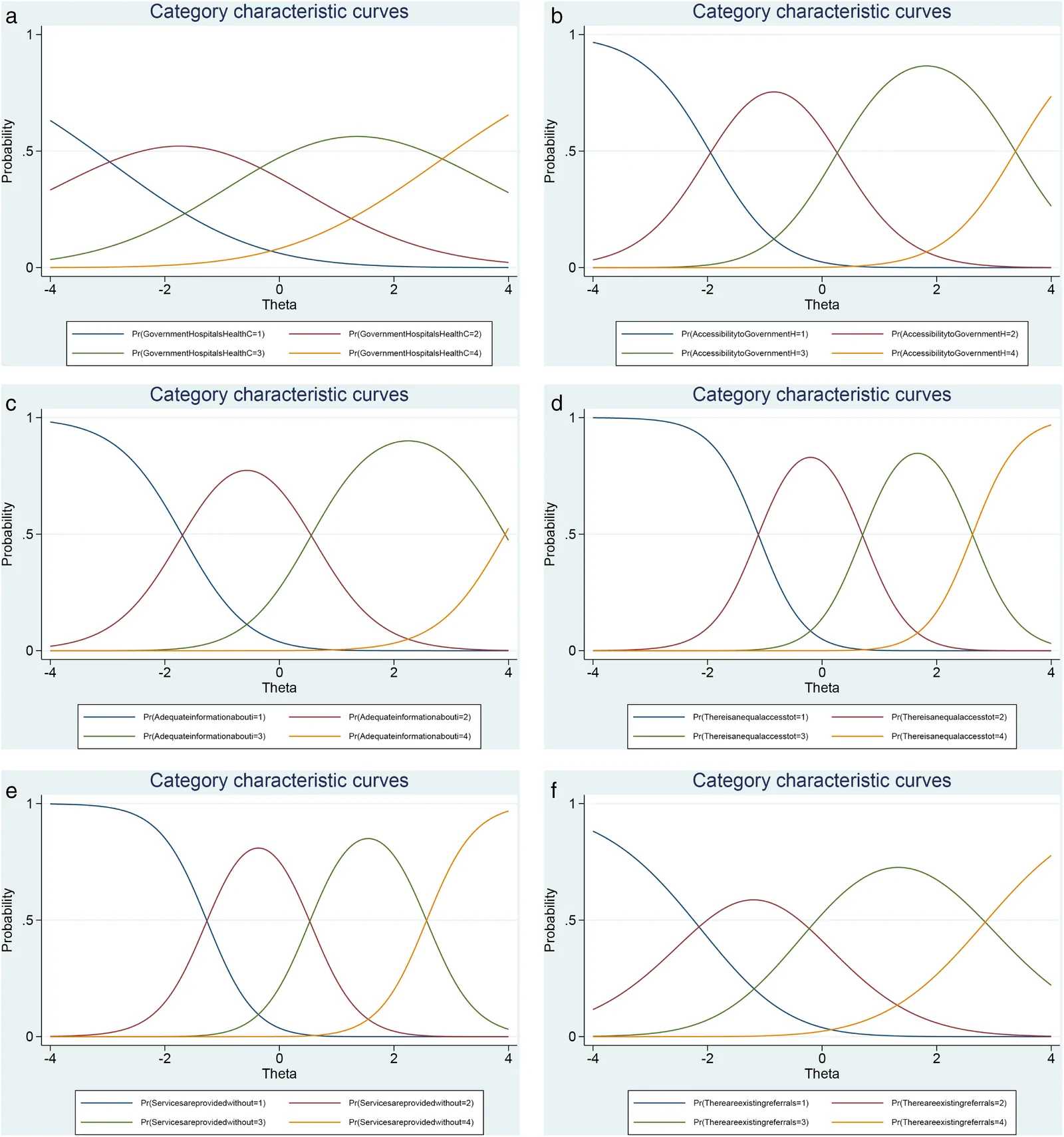
Category characteristic curves (CCC) for items 1 to 6. (a) Item 1: Availability of public healthcare, (b) Item 2: Access to public healthcare(c) Item 3: Adequate information services, (d) Item 4: Equal access(e) Item 5: Non-discrimination, (f) Item 6: Referral system. The x-axis represents the latent trait (θ), and the y-axis represents the probability of endorsing each response category.

**Fig 2:**
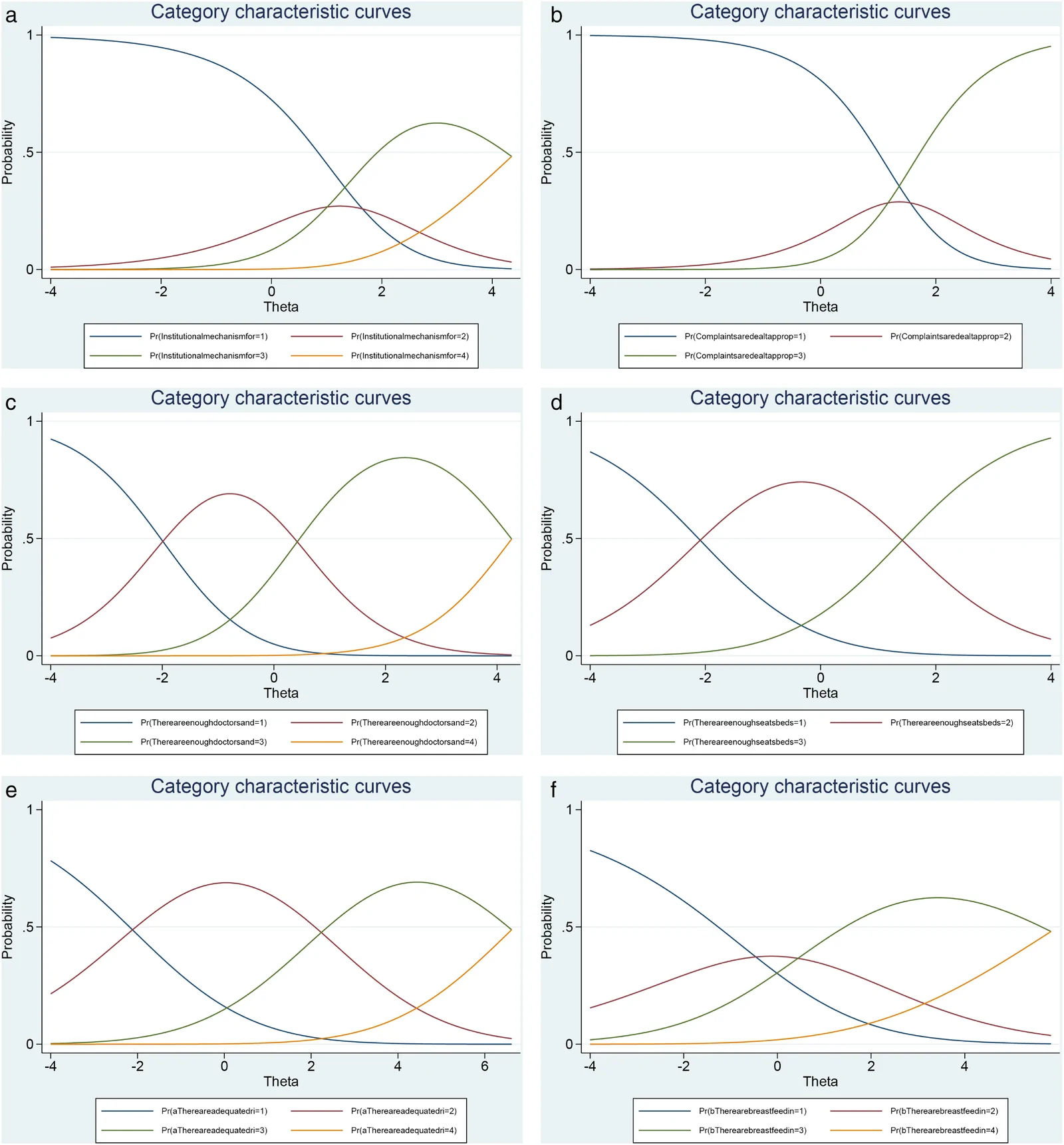
Category characteristic curves (CCC) for items 7 to 12. (a) Item 7: Grievance mechanism, (b) Item 8: Complaint handling(c) Item 9: Patient-doctor ratio, (d) Item 10: Number of in-patient beds(e) Item 11: Safe drinking water, (f) Item 12: Breastfeeding support. The x-axis represents the latent trait (θ), and the y-axis represents the probability of endorsing each response category.

**Fig 3:**
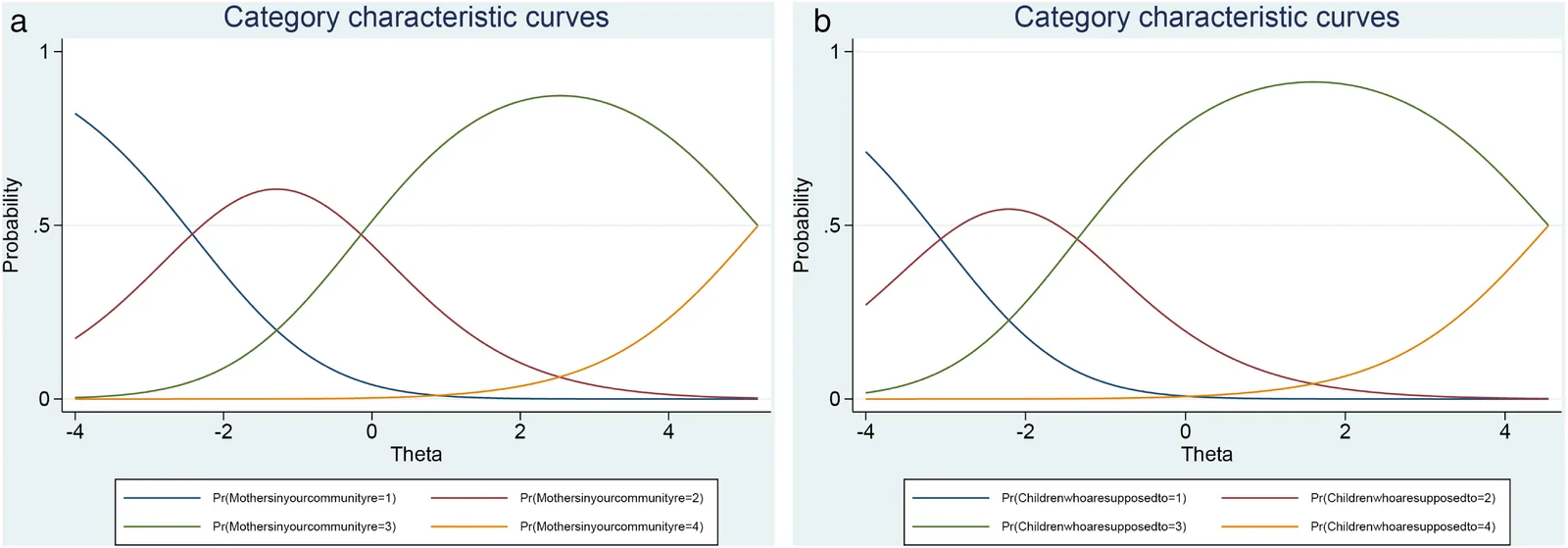
Category characteristic curves (CCC) for items 13 and 14. (a) Item 13: Mother care, (b) Item 14: Child care. The x-axis represents the latent trait (θ), and the y-axis represents the probability of endorsing each response category.

Category Characteristic Curves (CCCs) under the GPCM show well-separated response categories for the majority of items, with the probability of endorsing higher response categories increasing monotonically with higher levels of the latent trait. CCCs for items 1–6 are presented in Fig 1, those for items 7–12 in Fig 2, and those for items 13 and 14 in Fig 3. From a policy perspective, the consistency of monotonic ordering across the scale gives users a clear interpretation: a higher latent score corresponds reliably to higher endorsement of more favorable categories, which means that aggregated scorecard outputs can be used to rank or compare communities along a meaningful gradient of perceived quality. The visibly compressed curves for items where the highest category (4 = Good) was rare, such as complaint handling and number of in-patient beds, mirror the substantive finding that ‘Good’ service experiences are exceptional in this population.

Reading across the three figure panels, several specific patterns deserve more direct policy interpretation than a purely descriptive account would offer. The CCCs in Fig 1 for equal access (item 4), non-discrimination (item 5), and adequate information services (item 3) show cleanly separated category curves, with each category peaking at a distinct region of the latent trait. These are the items that carry the most measurement information. And the managers may treat as headline indicators when comparing performance across communities, tracking change over time, or constructing composite indices for resource allocation decisions. The fact that fairness and information items emerge as the most discriminating is itself a finding with policy weight: it suggests that community judgments of healthcare quality in this population are anchored more in equity and transparency than in any single facility characteristic, and that interventions targeting interpersonal aspects of care may move the scorecard needle faster than infrastructure-only investments.

At the other end of the spectrum, the CCC in Fig 2 for complaint handling (item 8) shows a sharply compressed structure in which the highest response category was never observed, and the curve for grievance mechanism (item 7) shows the ‘Good’ category emerging only at the extreme right tail of the latent trait. The practical reading is that institutional grievance and complaint mechanisms are not just rated badly; they are rated so uniformly badly that the scorecard, in practice, cannot distinguish between communities on the upper end of this domain. For policymakers, this is a strong signal that systemic reform of accountability mechanisms is required, rather than community-by-community improvement projects, and that any monitoring strategy may treat low scores on these two items as a chronic system-level finding rather than as a local outlier. Items capturing infrastructure conditions, including number of in-patient beds (item 10) and safe drinking water (item 11), show similarly compressed upper categories, pointing to facility-level investment needs that fall outside the immediate reach of community engagement activities.

A third set of curves, for breastfeeding support (item 12) and availability of public healthcare (item 1), shows more overlap among adjacent categories and weaker separation around the middle of the latent trait. The implication for routine monitoring is twofold. First, respondents may genuinely struggle to differentiate among intermediate response options for these items, in which case rewording or a move to a coarser two- or three-category response set may be appropriate in future revisions. Second, these items are less useful as standalone indicators and are best read alongside the more discriminating items in the same domain when constructing dashboards or scorecards intended to drive action. Taken together, the figures translate into a clear ordering of analytic uses: fairness and information items as headline indicators; grievance and infrastructure items as flags for systemic reform; and the less discriminating items as candidates for instrument refinement.

Fig 3 presents the Category Characteristic Curves for the two maternal and child health items (Mother care and Child care), demonstrating that higher perceived service quality is consistently associated with greater probability of endorsing more favorable response categories. The curves for Child care (Item 14) show particularly clean separation between categories, with a clear progression from lower to higher response options as the latent trait increases, while Mother care (Item 13) exhibits more gradual transitions, suggesting that respondents may differentiate less sharply between intermediate categories for this item. Both items contribute meaningfully to the overall scale, though their discrimination parameters are more moderate than those observed for fairness and access items.

### Test characteristic curve (TCC)

The Test Characteristic Curve (TCC) illustrates the relationship between θ and the expected total score on the 14-item scale. The expected test score increases monotonically with θ, indicating that FGDs with more favorable perceptions of healthcare services are associated with higher observed scores. The smooth, approximately linear progression across the central range of θ supports the interpretability of the summed score as a meaningful indicator of underlying healthcare service perceptions.

From a practical standpoint, the shape of the TCC has direct implications for how the summed scorecard score could be used. The approximately linear central segment of the curve means that, for the bulk of the population, equal differences in summed score correspond to equal differences in perceived service quality, so the summed score is a defensible tool for tier 1 prioritization decisions such as identifying communities that may be targeted first for service strengthening. At the lower extreme, however, the curve flattens, meaning that further reductions in summed score do not correspond to proportional reductions in perceived quality among the most disadvantaged communities; in practical terms, a community with a very low score may be functionally similar to one with a slightly higher very low score, and managers may not over-interpret small differences at this end. Equivalently, at the upper end, modest gains in summed score may understate substantive improvements in perception. For these reasons we recommend that summed scores be used for broad ranking and resource targeting, and that item-level diagnostics, especially on the more discriminating fairness and information items, be used to inform fine-grained operational decisions.

### Test information function (TIF)

The Test Information Function (TIF) showed that the scale provides the greatest measurement precision for respondents with low to moderate levels of perceived service quality, approximately within the range θ ∈ [−1.5, 1.0]. Precision declines at more extreme values of θ, particularly at very high levels of perceived service quality. This information profile has direct practical implications. Because marginalized populations in Bangladesh, as documented in the descriptive statistics, predominantly occupy the lower portion of the perceived quality continuum, the instrument is most precise exactly where it is most needed for equity monitoring. For policy use this means that the scorecard is well suited to detect deteriorations or modest improvements among the worst-served communities, and is therefore appropriate for impact evaluations of interventions targeting the floor of service quality. Conversely, the scale is less informative for tracking improvements among communities that already report relatively favorable experiences, and we would not recommend using it to benchmark high-performing settings without modification.

Several concrete uses follow from this profile. First, the instrument is appropriate as an early warning tool for routine surveillance of marginalized communities: a community already located at the floor of perceived quality will have any further deterioration measured with high precision, since that is exactly the range where the scale carries the most information. Second, the scale is well calibrated for impact evaluation of interventions aimed at the most underserved, where measurable improvement is likely to occur in the low to moderate range that the instrument captures with the highest precision. Third, for benchmarking among communities that already report relatively favorable experiences, the scorecard could be paired with complementary tools such as facility audits, clinical quality indicators, or independent client exit interviews, because the instrument on its own cannot reliably distinguish among communities in this range. Finally, the precision profile suggests a natural operational rule for monitoring dashboards: communities whose estimated θ falls below approximately −1.5 may trigger immediate programmatic review, while those between −1.5 and 1.0 can be ranked with confidence and tracked over successive measurement rounds. Read in this way, the TIF is not just a psychometric diagnostic but a guide to how the scorecard may be deployed within a national equity monitoring system.

### Summary of psychometric performance

Sampling adequacy was confirmed by a high KMO statistic (0.83) and a significant Bartlett’s test of sphericity. EFA with promax rotation identified three substantively meaningful and correlated dimensions: Accessibility and Fairness, Institutional Responsiveness, and Maternal and Child Health Support. Reliability was high (α = 0.84; ω = 0.87), and GPCM analysis demonstrated that items discriminated meaningfully and that response categories functioned in a substantially ordered manner. Taken together, these findings support the structural validity and internal consistency of the 14-item scale for use among marginalized populations in Bangladesh, while also identifying specific items and thresholds that warrant refinement and pointing to the need for confirmatory testing in independent samples.

## Discussion

This study set out to evaluate the psychometric properties of a 14-item Community Scorecard for measuring perceived healthcare service quality among marginalized populations in Bangladesh. By combining Classical Test Theory with the Generalized Partial Credit Model from Item Response Theory, the analysis goes beyond conventional scale validation and offers an item- and category-level picture of how the instrument behaves in this population. The findings reinforce the value of taking community perceptions seriously as a measurable element of health system performance, in line with broader calls for more responsive and equitable care [1,5,6].

The three-factor structure that emerges, with subdomains for Accessibility and Fairness, Institutional Responsiveness, and Maternal and Child Health Support, is consistent with multidimensional frameworks of healthcare quality in LMICs [1,22]. Communities appear to evaluate quality through interconnected lenses: whether they can access care without discrimination, whether institutions respond when something goes wrong, and whether supportive services exist for mothers and children [22,31,32]. Reliability indicators support the use of the full scale as a coherent measure, while remaining consistent with the presence of meaningful subdimensions [13,15].

The GPCM analysis added information that CTT alone cannot provide. Items related to equal access, non-discrimination, and information provision showed the highest discrimination, which is consistent with the view that judgments about fairness, transparency, and respectful communication weigh heavily in community evaluations of quality in marginalized settings [3,22,23]. Items related to specific facility conditions, including breastfeeding support, showed lower discrimination, suggesting that these may be less differentiating in the present context. The dominance of fairness-related items as drivers of measurement information echoes findings from the broader literature on health system trust [8,33].

Two major caution may be flagged before drawing stronger conclusions. First, although the GPCM was estimated under a unidimensional assumption, the underlying structure is multidimensional. We treated the scale as approximately unidimensional because the first factor was clearly dominant and because the small sample size limited what could be reliably estimated with a multidimensional IRT model, but we recognize that this is a working approximation rather than a strict claim. Future work in a larger sample would test a multidimensional or bifactor GPCM, formally examine local independence among the maternal and child health and grievance items, and consider hybrid scoring strategies that report both an overall index and subdomain scores. Second, the conclusion that response categories function ‘properly’ can be moderated. Most thresholds were ordered and statistically significant, which is encouraging, but several intermediate thresholds were not significantly estimated, indicating that some adjacent categories may not be clearly distinguished by respondents for those items. We therefore prefer to describe the response structure as substantially but not uniformly well functioning.

On substance, the results echo persistent concerns about Bangladesh’s health system [30,34]. The fact that the Accessibility and Fairness dimension shows the strongest discrimination identifies it as a primary lever for policy intervention, particularly investments in equitable resource allocation, expansion of the rural health workforce, and anti-discrimination policies [1,21]. The very low scores and high threshold parameters for the grievance and complaint handling items, in contrast, document a deep deficit in institutional accountability for the populations studied: formal feedback channels appear to be either inaccessible or ineffective for marginalized groups [11,35]. Tools like a validated CSC can contribute to closing this accountability gap, although the CSC itself is a measurement and dialogue mechanism rather than a substitute for system reform [11,24,36].

The peak measurement precision of the scale falls in the low to moderate range of the latent trait, which is exactly where marginalized populations in this study tend to be located. The instrument is therefore well targeted to monitor disparities and to evaluate interventions aimed at the most underserved [10,17,30,37], but it is less suited to benchmarking communities at the upper end of perceived quality.

It is important to weigh these strengths against several methodological constraints. The data come from a single cross-sectional round of CSCs, so the analysis cannot speak to whether perceptions change over time or in response to interventions. Each unit of analysis is a consensus rating from an FGD rather than an individual response, which is appropriate for a community scorecard but introduces the possibility that vocal participants disproportionately shape group ratings; this is a recognized property of consensus methods and warrants triangulation with individually administered surveys. The sample, while spanning all 64 districts and nine marginalized groups, was not drawn as a strictly probabilistic national sample; parameter estimates are therefore be treated as informative for the populations studied rather than as nationally representative. Social desirability and response biases are also plausible, especially given that the topics under discussion concern public services on which participants may have ongoing relationships with local officials. Finally, our work is an exploratory psychometric evaluation; we did not perform a separate confirmatory factor analysis or test the model in an independent validation sample, and the three-factor structure are therefore be regarded as a hypothesis to be confirmed in subsequent studies rather than as a definitively established structure.

### Limitations and future research

In light of the methodological complexity of this study, the limitations deserve a more developed treatment. We organize them under four headings.

**Sampling and unit of analysis:** The analytic unit is the FGD rather than the individual respondent. This is faithful to the Community Scorecard tradition but means that the data describe community-level perceptions rather than the distribution of opinion among individuals. Group consensus scoring can mask intra-group disagreement, and there is no guarantee that the consensus is unbiased with respect to the most marginalized members within each FGD. The sample also includes only one FGD per community, which limits checks of within-community reliability. The selection of five marginalized communities per district was random within a curated frame, but the frame itself depended on local lists maintained by BRAC and partners and may underrepresent groups that are particularly invisible to local organizations.

**Ethical oversight:** As described in the Ethics section, the study did not undergo prospective review by an external Institutional Review Board, and oversight was exercised through internal governance procedures at BRAC and CRD. This is a limitation for the international transferability of the findings, even though informed consent and confidentiality protections were observed. We recommend that future cycles of the CSC research program be submitted for prospective IRB review.

**Model assumptions:** The GPCM was estimated under a unidimensional assumption that was empirically defensible but not strictly correct given the multidimensional CTT solution. was inspected and the most notable residual correlations were modest, but residuals among the two grievance items and among the two maternal items are consistent with the documented subdomain structure and could bias item parameters somewhat. The use of an oblique rotation and our retention thresholds are standard choices, but other reasonable choices (for example, varimax rotation or a stricter loading threshold) would yield slightly different solutions. The absence of confirmatory factor analysis in an independent sample is a particular limitation.

**Response bias and measurement scope:** Self-reported perceptions are sensitive to recall and social desirability biases. The scale focuses on perceived service quality; it does not measure objective service quality, clinical outcomes, or structural inputs. The fact that no FGD endorsed the top category on any item is consistent with genuine dissatisfaction but might also reflect a cultural reluctance to give the highest praise to government services, which is a hypothesis that future cognitive interviewing could test.

Future research may employ longitudinal designs to assess sensitivity to change, perform confirmatory factor analysis in an independent sample, test multidimensional and bifactor IRT specifications, refine items flagged for high uniqueness or weak intermediate thresholds through cognitive interviewing, and link community perception scores from this instrument with objective clinical and structural quality metrics to build a multi-method evidence base [1,15,38].

## Conclusion

This study evaluates a 14-item Community Scorecard as an instrument for measuring perceived healthcare service quality among marginalized populations in Bangladesh, combining Classical Test Theory and the Generalized Partial Credit Model. The evidence indicates that the scale is internally consistent, has a substantively meaningful three-factor structure, and shows useful item- and category-level performance for the populations studied. We are more confident that the instrument is suitable for monitoring perceived quality in low to moderate ranges than that it is broadly generalizable; confirmatory testing in independent samples, refinement of items with high uniqueness or weak intermediate thresholds, and a multidimensional IRT modeling exercise in a larger sample would all strengthen the basis for routine policy use.

Substantively, the findings highlight perceived inequities in access and fairness, and a particularly sharp deficit in institutional responsiveness to community concerns. These patterns are consistent with the broader literature on marginalized populations in Bangladesh and warrant attention from policymakers, health managers, and civil society. However, as features of perceived service quality among the populations and FGDs sampled in this study rather than as definitive national findings.

Used appropriately and alongside complementary data sources, the validated scorecard can contribute to a more inclusive and accountable approach to monitoring health systems in Bangladesh and similar contexts.

## Supporting information

S1 File FGD checklist in Bengali. The Community Scorecard focus group discussion checklist as administered in the native language.
(PDF)

S2 File FGD checklist/questionnaire in English. English version of the 14-item Community Scorecard instrument and accompanying discussion guide.
(PDF)

S3 File Data. Community-level (FGD) item scores for the 311 scoring units used in all analyses.
(XLS)
