# Supplementary material for: Psychometric validation of a community healthcare scorecard used for marginalized populations in Bangladesh
Source: PLoS One. 2026 Aug 24;21(8):e0348720. doi: 10.1371/journal.pone.0348720 (PMC13502686; doi:10.1371/journal.pone.0348720)
Supplement: Supplementary file 1 [file pone.0348720.s001.pdf]

P37_Community Scorecard (CSC)_ BRAC

স্বাস্থ্যসেবা, শিক্ষা ও সামাজিক নিরাপত্তা বিষয়ক এফজিডি /কমিউনিটি স্কোর কার্ড, ২০২৩

আমি_। আমি সিআরডি নামক ঢাকার একটি গবেষণা প্রতিষ্ঠানে কাজ করি। সিআরডি, ব্র্যাক বাংলাদেশ কর্তৃক প্রান্তিক জনগোষ্ঠীর মানুষদের বিভিন্ন পরিষেবার মূল্যায়ন এর একটি গবেষণা কাজ হাতে নিয়েছে। এই গবেষণার জন্য আমরা প্রান্তিক জনগোষ্ঠীর মানুষদের সাথে কমিউনিটি স্কোর কার্ড এবং এফজিডি করছি। আজকে আমরা আপনার সাথে একটি কমিউনিটি স্কোর কার্ড ও এফজিডি করতে ইচ্ছুক!

অংশগ্রহণকারী নির্ণয়ের ক্ষেত্রে নির্দেশাবলি • আলোচনার জন্য সময়: ৬০-৯০ মিনিট অংশগ্রহণকারীদেরকে বিভিন্ন প্রান্তিক জনগোষ্ঠীর মানুষদের মধ্য থেকে নির্বাচন করতে হবে। উত্তরদাতাদের অন্তর্বিভাগীয় শ্রেণীবিভাগ নিশ্চিত করতে বিভিন্ন রকমের বয়স, শিক্ষা, পেশা ইত্যাদি নিশ্চিত করতে হবে।

এফজিডি/ স্কোর কার্ড পরিচালনার জন্য কিছু পরামর্শঃ 1. আলোচনা শুরু করার পূর্বে আলোচনার সময় সম্পর্কে অংশগ্রহণকারীদের অবগত করতে হবে। 2. অনুগ্রহ করে নিজের পরিচয় দিয়ে এবং সময়মতো আলোচনা শুরু করুন। 3. অনুগ্রহ করে আলোচনার জন্য সঠিক লোকদের ব্যবস্থা করা হয়েছে কিনা যাচাই করুন। 4. অনুগ্রহ করে অংশগ্রহণকারীদের স্বাক্ষর এবং বিবরণ সঠিকভাবে নথিভুক্ত করা হয়েছে কিনা নিশ্চিত করুন। 5. নোট নেওয়া এবং ভয়েস রেকর্ড করার জন্য অংশগ্রহণকারীদের কাছ থেকে অনুমতি গ্রহন করুন। 6. অংশগ্রহণকারীদের প্রয়োজনীয় উদাহরণ এবং উৎসাহ প্রদান করুন যাতে তারা ইচ্ছাকৃতভাবে আলোচনায় অংশগ্রহণ করে। 7. অবিলম্বে প্রকাশিত প্রতিক্রিয়া যাচাই করার জন্য কমপক্ষে ২-৩ জন অংশগ্রহণকারীর অংশগ্রহণ নিশ্চিত করুন। 8. উদাহরণ দেওয়ার সময় এবং আলোচনায় অংশগ্রহণ করার সময় বা আলোচনার জন্য বসার ব্যবস্থা করার সময় স্থানীয় সংস্কৃতি এবং ধর্মীয় সংবেদনশীলতা বিবেচনা করুন। 9. অনুগ্রহ করে প্রত্যেকেরই নিজ নিজ মতামত বা অভিজ্ঞতা শেয়ার করার সময় সমান সুযোগ নিশ্চিত করুন। 10. আলোচনা শুরু করার আগে, অনুগ্রহ করে আলোচনায় অপরিচিতদের প্রবেশ/আলোচনায় বাধা দেওয়া রোধ করতে একজনের ভূমিকা পালন নিশ্চিত করুন। 11. বসার ব্যবস্থা COVID-19 স্বাস্থ্য সুরক্ষা প্রোটোকলের সাথে সঙ্গতিপূর্ণ (দূরত্ব, সমস্ত অংশগ্রহণকারীদের মাস্ক পরা, তাপমাত্রা পরীক্ষা ইত্যাদি) নিশ্চিত করুন।

কমিউনিটি স্কোর কার্ড (CSC): কমিউনিটি স্কোর কার্ড (CSC) একটি অংশগ্রহণমূলক, কমিউনিটি ভিত্তিক পর্যবেক্ষণ এবং মূল্যায়ন টুল যা দিয়ে নাগরিকদের স্বাস্থ্যকেন্দ্র, স্কুল, গণপরিবহন, জল, বর্জ্যের মতো জনসেবার গুণগত মূল্যায়ন নিশ্চিত করতে সাহায্য করে।

লাইকার্ট স্কেল: সামাজিক বিজ্ঞানে মানুষের ব্যবহার বা তাদের চারিত্রিক বিভিন্ন বৈশিষ্ট্য কিংবা বা তাদের কোন কিছুর সম্ভাব্যতা, আগ্রহ, ইচ্ছা এরকম অনেক বিষয়গুলোই পরিমাপ করা হয়। লাইকার্ট এক ধরনের রেটিং স্কেল। উক্ত গবেষণাতে এই রেটিং স্কেলটি ব্যবহার করে আমরা কমিউনিটি স্কোরকার্ডটি করব । স্কোরকার্ডের রেটিং এ তাদের যে রেসপন্স নেয়া হবে তা অবশ্যই প্রশ্নপত্রের মাধ্যমে মতামত গ্রহন করে, তা র‍্যাংকিং করা হবে সর্বোচ্চ (৫) থেকে সর্বনিম্ন (১) বা পজিটিভ (৫)থেকে নেগেটিভ (১) বিভিন্ন ক্যাটাগরিতে তাদের মতামত এর রেটিং অনুযায়ী৷ এসবের মাধ্যমে বোঝা যায় বিষয়টা কতটা গুরুত্বপূর্ণ . এই স্কেল এ একটা অপশন বাছাই করতে হবে উত্তরদাতাকে। এটি সব সময় ই নির্বাচনীমূলক প্রশ্ন অর্থাৎ এতে কোন ব্যখ্যা দেয়ার সুযোগ থাকবে উত্তরদাতার৷ অপশনসমুহ: ১- খুব খারাপ, ২- খারাপ, ৩- মোটামোটি , ৪- ভালো, ৫- খুব ভালো

১। আপনারা কি কমিউনিটি স্কোর কার্ড ও এফজিডি অংশগ্রহন করতে ইচ্ছুক?

- ○ হ্যা
- ○ না

২। কমিউনিটি টি যে বিভাগে অবস্থিত?

- ঢাকা
- সিলেট
- চট্টগ্রাম
- রাজশাহী
- খুলনা
- বরিশাল
- রংপুর
- ময়মনসিংহ

২.১ কমিউনিটি টি যে জেলায় অবস্থিত?

- ঢাকা
- মানিকগঞ্জ
- নরসিংদী
- মুন্সিগঞ্জ
- রাজবাড়ী
- গোপালগঞ্জ
- মাদারীপুর
- ফরিদপুর
- শরীয়তপুর
- নারায়ণগঞ্জ
- টাঙ্গাইল
- কিশোরগঞ্জ
- গাজীপুর

২.১ কমিউনিটি টি যে জেলায় অবস্থিত?

- সিলেট
- মৌলভীবাজার
- হবিগঞ্জ
- সুনামগঞ্জ

২.১ কমিউনিটি টি যে জেলায় অবস্থিত?

- ফেনী
- খাগড়াছড়ি
- চট্টগ্রাম
- লক্ষ্মীপুর
- কক্সবাজার
- চাঁদপুর
- নোয়াখালী
- রাঙ্গামাটি
- কুমিল্লা
- বান্দরবান
- ব্রাহ্মণবাড়িয়া

২.১ কমিউনিটি টি যে জেলায় অবস্থিত?

- সিরাজগঞ্জ,
- নওগাঁ
- চাঁপাইনবাবগঞ্জ
- জয়পুরহাট
- নাটোর
- বগুড়া
- রাজশাহী
- পাবনা

২.১ কমিউনিটি টি যে জেলায় অবস্থিত?

- ঝিনাইদহ
- বাগেরহাট
- খুলনা
- যশোর
- চুয়াডাঙ্গা
- কুষ্টিয়া
- মাগুরা
- নড়াইল
- সাতক্ষীরা
- মেহেরপুর

২.১ কমিউনিটি টি যে জেলায় অবস্থিত?

- ঝালকাঠি
- বরগুনা
- ভোলা
- বরিশাল
- পিরোজপুর
- পটুয়াখালী

২.১ কমিউনিটি টি যে জেলায় অবস্থিত?

- পঞ্চগড়
- দিনাজপুর
- লালমনিরহাট
- নীলফামারী
- গাইবান্ধা
- ঠাকুরগাঁও
- রংপুর
- কুড়িগ্রাম

২.১ কমিউনিটি টি যে জেলায় অবস্থিত?

- শেরপুর
- ময়মনসিংহ
- জামালপুর
- নেত্রকোণা

২.২ কমিউনিটি টি যে উপজেলায় অবস্থিত?

____

২.৩ কমিউনিটি টি যে ইউনিয়নে অবস্থিত?

____

২.৪ কমিউনিটি টি যে গ্রামে অবস্থিত?

____

৩ এফজিডি কোড - বিভাগ

- ঢাকা
- সিলেট
- বরিশাল
- খুলনা
- রংপুর
- ময়মনসিংহ
- রাজশাহী
- চট্টগ্রাম

কোড নাম্বার

1 20

কোড নাম্বার

21 85

কোড নাম্বার

86 115

কোড নাম্বার

116 135

কোড নাম্বার

136 175

কোড নাম্বার

176 215

কোড নাম্বার

216 270

কোড নাম্বার

271 320

৪ মোট অংশগ্রহণকারির সংখ্যা

____

৪.১ অংশগ্রহনকারির ধরণ

- নারী
- পুরুষ

৫ এলাকার ধরণ

- ◯ শহর (সিটি কর্পোরেশন/পৌরসভা)
- ◯ গ্রাম

৬.১ ইসলাম ধর্মাবলম্বি ?

____

৬.২ হিন্দু ধর্মাবলম্বি ?

____

৬.৩ বৌদ্ধ ধর্মাবলম্বি ?

____

৬.৪ খ্রিষ্টান ধর্মাবলম্বি ?

____

৬.৫ অন্যান্য

____

৭.১ বয়সসীমা ১৮-৪০ বছর

____

৭.২ বয়সসীমা ৪১-৬০ বছর

____

৭.৩ বয়সসীমা ৬০+ বছর

____

সেকশন এ
প্রাথমিক তথ্যাবলি

৮ অংশগ্রহণকারীরা কোন কমিউনিটির?

- দলিত
- ক্ষুদ্র জাতিসত্তা/আদিবাসী
- প্রতিবন্ধী ব্যক্তি
- ট্রান্সজেন্ডার/তৃতীয় লিঙ্গ
- যৌনকর্মী
- অভ্যন্তরীন বাস্তুচ্যুত/বাস্তুচ্যুত জনগোষ্ঠী
- ভিক্ষাবৃত্তিতে জড়িত বয়স্ক ব্যক্তি
- শহরের ভাসমান মানুষ
- বেদে
- দুর্গম এলাকায় বসবাসরত হতদরিদ্র মানুষ (চর, হাওর, পাহাড়, দ্বীপ)

৯ অংশগ্রহণকারীদের পেশা (একাধিক উত্তর)

- [ ] কৃষিকর্ম/মৎস্য চাষ/ শুকর চরানো/ গরুছাগল চরানো
- [ ] জেলে
- [ ] মুচি
- [ ] কামার/ কুমার/ তাঁতি/ কুটিরশিল্প/হস্তশিল্প
- [ ] রাজমিস্ত্রি/ কাঠমিস্ত্রি/ মেকানিক
- [ ] দিনমজুর/ ক্ষেতমজুর/ শ্রমিক
- [ ] রিকশা/ভ্যান চালক
- [ ] পরিবহনচালক
- [ ] পোশাকশ্রমিক
- [ ] পরিচ্ছন্নতাকর্মী
- [ ] ক্ষুদ্র ব্যবসা (মূলধন ১০হাজারের কম)
- [ ] মাঝারি ব্যবসা (১০হাজার থেকে ১ লাখ)
- [ ] বৃহৎ ব্যবসা (১ লাখ এর বেশি)
- [ ] সরকারি চাকরি
- [ ] বেসরকারি চাকরি
- [ ] গৃহিণী
- [ ] শিক্ষক
- [ ] নার্স
- [ ] অন্যান্য স্বাস্থ্যকর্মী
- [ ] জনপ্রতিনিধি
- [ ] ইমাম/পুরোহিত/ ধর্মগুরু
- [ ] বেকার
- [ ] শিক্ষার্থী
- [ ] এনজিওকর্মী
- [ ] যৌনকর্মী
- [ ] ভিক্ষাবৃত্তি
- [ ] গৃহকর্মী
- [ ] অন্যান্য

সেকশন বি:

সরকারি চিকিৎসা সেবায় অন্তর্ভুক্তি

---

১০ সরকারি হাসপাতাল/স্বাস্থ্যকেন্দ্র আপনাদের এলাকার কাছাকাছি (সহজেই হাসপাতাল/স্বাস্থ্য কেন্দ্রে যাতায়াত করা যায়)	৫- খুব ভালো	৪- ভালো	৩-মোটামোটি	২- খারাপ	১- খুব খারাপ
দলের মতামত দিন	○	○	○	○	○
১১সরকারি হাসপাতাল/স্বাস্থ্য কেন্দ্রে প্রবেশগম্যতা (সেবা নিতে গেলে সেবা পাওয়া যায়)	৫- খুব ভালো	৪- ভালো	৩-মোটামোটি	২- খারাপ	১- খুব খারাপ
দলের মতামত দিন?	○	○	○	○	○
১২ স্বাস্থ্য বিষয়ে পর্যাপ্ত অন্তর্ভুক্তিমূলক তথ্য (তথ্যসমূহ আপনাদের জন্য বোধগম্য)	৫- খুব ভালো	৪- ভালো	৩-মোটামোটি	২- খারাপ	১- খুব খারাপ
দলের মতামত দিন?	○	○	○	○	○
১৩ সরকারি হাসপাতাল/স্বাস্থ্য কেন্দ্রে চিকিৎসার সমান সুযোগ বিদ্যমান (আপনাদের সাথে কোনো প্রকার ঔষধপত্র অথবা ডাক্তার এর সাথে দেখা করার ক্ষেত্রে বৈষম্য করা হয় না)	৫- খুব ভালো	৪- ভালো	৩-মোটামোটি	২- খারাপ	১- খুব খারাপ
দলের মতামত দিন?	○	○	○	○	○
১৪ সরকারি হাসপাতালে অবহেলা ছাড়াই সেবা প্রদান করা হয় (সেবা দিতে আপনাদের সাথে খারাপ ব্যবহার, ঘৃণা-তুচ্ছ-তাচ্ছিল্য ইত্যাদি করা হয় না)	৫- খুব ভালো	৪- ভালো	৩-মোটামোটি	২- খারাপ	১- খুব খারাপ
দলের মতামত দিন?	○	○	○	○	○
১৫ সরকারি হাসপাতালে/স্বাস্থ্য কেন্দ্রে রেফারেল ব্যবস্থা বিদ্যমান (সেবা দিতে সক্ষম না হলে সংশ্লিষ্ট রোগের যথাযথ চিকিৎসা হবে এমন হাসপাতালে যথাযথ প্রক্রিয়ায় প্রেরণ করা হয়)	৫- খুব ভালো	৪- ভালো	৩-মোটামোটি	২- খারাপ	১- খুব খারাপ
দলের মতামত দিন?	○	○	○	○	○
১৬ সরকারি হাসপাতালে অভিযোগ প্রদানের প্রাতিষ্ঠানিক ব্যবস্থা বিদ্যমান (সেবার মান সম্পর্কে অভিযোগ করা যায়)	৫- খুব ভালো	৪- ভালো	৩-মোটামোটি	২- খারাপ	১- খুব খারাপ
দলের মতামত দিন?	○	○	○	○	○
১৭ অভিযোগ করলে যথাযথ ব্যবস্থা গ্রহণ করা হয় (প্রতিকার করা হয়)	৫- খুব ভালো	৪- ভালো	৩-মোটামোটি	২- খারাপ	১- খুব খারাপ
দলের মতামত দিন?	○	○	○	○	○
১৮ আপনাদের সবচেয়ে কাছের সরকারি হাসপাতাল বা স্বাস্থ্য কেন্দ্রটিতে পর্যাপ্ত ডাক্তার ও নার্স রয়েছে	৫- খুব ভালো	৪- ভালো	৩-মোটামোটি	২- খারাপ	১- খুব খারাপ
দলের মতামত দিন?	○	○	○	○	○
১৯ আপনাদের সবচেয়ে কাছের সরকারি হাসপাতাল বা স্বাস্থ্য কেন্দ্রটিতে পর্যাপ্ত সিট/ বেড, সরঞ্জাম ও ওষুধের ব্যবস্থা আছে	৫- খুব ভালো	৪- ভালো	৩-মোটামোটি	২- খারাপ	১- খুব খারাপ
দলের মতামত দিন?	○	○	○	○	○

২০.১ আপনাদের সবচেয়ে কাছের সরকারি হাসপাতাল বা স্বাস্থ্য কেন্দ্রটিতে পর্যাপ্ত খাবার পানি, টয়লেট সুবিধা ইত্যাদি আছে

	৫- খুব ভালো	৪- ভালো	৩-মোটামোটি	২- খারাপ	১- খুব খারাপ
দলের মতামত দিন?	○	○	○	○	○

২০.২ আপনাদের সবচেয়ে কাছের সরকারি হাসপাতাল বা স্বাস্থ্য কেন্দ্রটিতে ব্রেস্ট ফিডিং কর্নার আছে

	৫- খুব ভালো	৪- ভালো	৩-মোটামোটি	২- খারাপ	১- খুব খারাপ
দলের মতামত দিন?	○	○	○	○	○

২১ গর্ভবতী মায়েদের সরকার থেকে যেসব সেবা পাওয়ার কথা (টিকা, ফলিক এসিড/ ভিটামিন, স্বাস্থ্যকর্মীর মাকে দেখতে আসা, ইত্যাদি) আপনাদের পাড়ায় মায়েদের সেসব সেবা দেওয়া হয়

	৫- খুব ভালো	৪- ভালো	৩-মোটামোটি	২- খারাপ	১- খুব খারাপ
দলের মতামত দিন?	○	○	○	○	○

২২ শিশুদের সরকার থেকে যেসব সেবা পাওয়ার কথা (টিকা, ৫ বছর পর্যন্ত বিনামূল্যে স্বাস্থ্য সেবা, ইত্যাদি) আপনাদের পাড়ায় শিশুদের সেসব সেবা দেওয়া হয়

	৫- খুব ভালো	৪- ভালো	৩-মোটামোটি	২- খারাপ	১- খুব খারাপ
দলের মতামত দিন?	○	○	○	○	○

২৩ আপনাদের সবচেয়ে কাছের সরকারি হাসপাতাল বা স্বাস্থ্য কেন্দ্রটিতে যেসব সেবা দেওয়া হয় সেগুলো মানসম্মত

	৫- খুব ভালো	৪- ভালো	৩-মোটামোটি	২- খারাপ	১- খুব খারাপ
দলের মতামত দিন?	○	○	○	○	○

সেকশন সি:

সরকারি প্রাথমিক শিক্ষায় অন্তর্ভুক্তি

---

২৪ প্রাথমিক বিদ্যালয় আপনাদের বাড়ি থেকে কাছেই/ সহজেই স্কুলে যাতায়াত করা যায়

	৫- খুব ভালো	৪- ভালো	৩-মোটামোটি	২- খারাপ	১- খুব খারাপ
দলের মতামত দিন?	○	○	○	○	○

২৫ আপনাদের সন্তান যে বিদ্যালয়ে যায় সেখানকার ক্লাসরুমে/শ্রেণীকক্ষে, খেলার মাঠে, পরীক্ষার হলে শিক্ষকরা আপনাদের সন্তানদের সাথে কোনো বৈষম্য করেন না

	৫- খুব ভালো	৪- ভালো	৩-মোটামোটি	২- খারাপ	১- খুব খারাপ
দলের মতামত দিন?	○	○	○	○	○

২৬ প্রাথমিক বিদ্যালয়টিতে শিক্ষার মান বা আনুষঙ্গিক বিষয়ে অভিযোগ করার সুযোগ আছে

	৫- খুব ভালো	৪- ভালো	৩-মোটামোটি	২- খারাপ	১- খুব খারাপ
দলের মতামত দিন?	○	○	○	○	○

২৭ অভিযোগ করলে যথাযথ ব্যবস্থা গ্রহণ করা হয় বা প্রতিকার পাওয়া যায়

	৫- খুব ভালো	৪- ভালো	৩-মোটামোটি	২- খারাপ	১- খুব খারাপ
দলের মতামত দিন?	○	○	○	○	○

২৮ প্রাথমিক বিদ্যালয়টিতে পর্যাপ্ত শিক্ষক/ শিক্ষিকা আছে

	৫- খুব ভালো	৪- ভালো	৩-মোটামোটি	২- খারাপ	১- খুব খারাপ

দলের মতামত দিন?					
২৯ প্রাথমিক বিদ্যালয়টিতে শিক্ষকদের পাঠদান বোধগম্য (ছেলে -মেয়েরা ক্লাসের পড়ালেখা বোঝে)	৫- খুব ভালো	৪- ভালো	৩-মোটামোটি	২- খারাপ	১- খুব খারাপ
দলের মতামত দিন?					
৩০ প্রাথমিক বিদ্যালয়টিতে শিক্ষার্থীদের ঝরে পড়ার হার কমানোর জন্য ব্যবস্থা গ্রহণ করা হয় (শিক্ষক হোম ভিজিট করেন)	৫- খুব ভালো	৪- ভালো	৩-মোটামোটি	২- খারাপ	১- খুব খারাপ
দলের মতামত দিন?					
৩১ কোভিড-পরবর্তী সময়ে শিক্ষার্থীদের প্রাথমিক স্কুলে ফিরিয়ে আনতে শিক্ষকদের পক্ষ থেকে যথেষ্ঠ উদ্যোগ ছিল	৫- খুব ভালো	৪- ভালো	৩-মোটামোটি	২- খারাপ	১- খুব খারাপ
দলের মতামত দিন?					
৩২ আপনারা অভিভাবক হিসেবে বিদ্যালয়টির অভিভাবক-শিক্ষক মিটিং-এ অংশগ্রহণ করতে পারেন	৫- খুব ভালো	৪- ভালো	৩-মোটামোটি	২- খারাপ	১- খুব খারাপ
দলের মতামত দিন?					
৩৩ আপনাদেরকে বিদ্যালয়টির অভিভাবক-শিক্ষক মিটিং-এ কথা বলার সুযোগ দেওয়া হয়	৫- খুব ভালো	৪- ভালো	৩-মোটামোটি	২- খারাপ	১- খুব খারাপ
দলের মতামত দিন?					
৩৪ প্রাথমিক শিক্ষা প্রতিষ্ঠানে যে শিক্ষা প্রদান করা হয় তা কি মানসম্মত?	৫- খুব ভালো	৪- ভালো	৩-মোটামোটি	২- খারাপ	১- খুব খারাপ
দলের মতামত দিন?					

সেকশন ডিঃ সামাজিক নিরাপত্তা প্রোগ্রামে অন্তর্ভুক্তি

সামাজিক নিরাপত্তা প্রোগ্রাম (যেমন বয়স্ক ভাতা, খাদ্য ও অর্থ সহায়তা, কাজের বিনিময়ে খাদ্য, মাতৃত্বকাল ভাতা, ওএমএস, সরকারি শিক্ষা বৃত্তি, মুক্তিযোদ্ধা ভাতা ইত্যাদি) বিষয়ে আপনাদের মূল্যায়ন কি?

---

৩৫ যে কোনো সামাজিক নিরাপত্তা কর্মসূচির (যেমন বয়স্ক ভাতা, খাদ্য ও অর্থ সহায়তা, মাতৃত্বকাল ভাতা, ইত্যাদি) জন্য যোগ্য হলে এই সেবা সহজেই পাওয়া যায়	৫- খুব ভালো	৪- ভালো	৩-মোটামোটি	২- খারাপ	১- খুব খারাপ
দলের মতামত দিন?					
৩৬ প্রকাশ্যে সভা ডেকে এসব সামাজিক নিরাপত্তা ভাতা/কার্ডের জন্য উপকারভোগী নির্বাচন করা হয়	৫- খুব ভালো	৪- ভালো	৩-মোটামোটি	২- খারাপ	১- খুব খারাপ
দলের মতামত দিন?					
৩৭ স্থানীয় জনপ্রতিনিধি ও সরকারি প্রতিনিধিদের কাছে গেলে সামাজিক নিরাপত্তা ভাতা/কার্ড বিষয়ে তথ্য পাওয়া যায়	৫- খুব ভালো	৪- ভালো	৩-মোটামোটি	২- খারাপ	১- খুব খারাপ
দলের মতামত দিন?					
৩৮ সামাজিক নিরাপত্তা ভাতা/কার্ড বিষয়ে যে তথ্য দেওয়া হয় তা বোধগম্য	৫- খুব ভালো	৪- ভালো	৩-মোটামোটি	২- খারাপ	১- খুব খারাপ

দলের মতামত দিন?	○	○	○	○	○
৩৯ সামাজিক নিরাপত্তা ভাতা/কার্ড প্রদানের জন্য উপকারভোগী নির্বাচনের ক্ষেত্রে (আপনাদের প্রতি) কোনো বৈষম্য করা হয় না	৫- খুব ভালো	৪- ভালো	৩-মোটামোটি	২- খারাপ	১- খুব খারাপ
দলের মতামত দিন?	○	○	○	○	○
৪০ উপকারভোগী (ভাতা পাওয়ার জন্য) নির্বাচনের ক্ষেত্রে স্বজনপ্রীতি, দলীয় লোকপ্রীতি, ভোট ব্যাংক বৃদ্ধি করা, ইত্যাদি করা হয় না	৫- খুব ভালো	৪- ভালো	৩-মোটামোটি	২- খারাপ	১- খুব খারাপ
দলের মতামত দিন?	○	○	○	○	○
৪১ সামাজিক নিরাপত্তা ভাতা/কার্ড নিতে কোনো প্রকার অর্থ (ঘুষ বা নিয়ম বহির্ভূত অর্থ) দিতে হয় না	৫- খুব ভালো	৪- ভালো	৩-মোটামোটি	২- খারাপ	১- খুব খারাপ
দলের মতামত দিন?	○	○	○	○	○
৪২ যে পরিমাণ টাকা, চাল, ইত্যাদি দেওয়ার কথা থাকে তা সঠিক পরিমাণে দেওয়া হয়	৫- খুব ভালো	৪- ভালো	৩-মোটামোটি	২- খারাপ	১- খুব খারাপ
দলের মতামত দিন?	○	○	○	○	○
৪৩ ৯. যেসব উপকরণ (চাল/গম/তেল) দেওয়া হয় সেগুলো মানসম্মত	৫- খুব ভালো	৪- ভালো	৩-মোটামোটি	২- খারাপ	১- খুব খারাপ
দলের মতামত দিন?	○	○	○	○	○
৪৪ যোগ্য হওয়া সত্ত্বেও সামাজিক নিরাপত্তা ভাতা/কার্ড না পেলে অথবা উপকরণ মানসম্মত না হলে অভিযোগ করা যায়	৫- খুব ভালো	৪- ভালো	৩-মোটামোটি	২- খারাপ	১- খুব খারাপ
দলের মতামত দিন?	○	○	○	○	○
৪৫ অভিযোগ করলে যথাযথ ব্যবস্থা গ্রহণ করা হয় বা প্রতিকার পাওয়া যায়	৫- খুব ভালো	৪- ভালো	৩-মোটামোটি	২- খারাপ	১- খুব খারাপ
দলের মতামত দিন?	○	○	○	○	○

সেকশন ইঃ

সঞ্চালক দলের পরিচয়

---

৪৬ সুপারভাইজরের নাম

○ মোঃ আনোয়ারুল ইসলাম

৪৭ সুপারভাইজরের মোবাইল নাম্বার

○ 01719268622

৪৮ সুপারভাইজরের ই-মেইল এড্রেস

○ anowarul@crdbd.org

৪৯ মনিটরিং অফিসারের নাম

- মোঃ আনোয়ারুল ইসলাম
- মাহমুদা আক্তার
- তন্ময় নাথ
- সুমাইয়া আক্তার
- মোঃ আরাফাত ইসলাম
- রাহাত হান্নান
- নাজিয়া নাজনীন

৫০ মনিটরিং অফিসারে মোবাইল নাম্বার

- 01719268622
- 01731088022
- 01873491169
- 01885554108
- 01719027250
- 01521322486
- 01687620675

৫১ মনিটরিং অফিসারে ই-মেইল এড্রেস

- anowarul@crdbd.org
- anna@crdbd.org
- tonmoy@crdbd.org
- sumaiya@crdbd.org
- arafat@crdbd.org
- rahat@crdbd.org
- nazia@crdbd.org

৫২ গবেষণা সহকারির (১) নাম

- ◯ সাদিয়া প্রীতি
- ◯ জেসমিন আক্তার
- ◯ জামাল উদ্দিন মিয়া
- ◯ ইকবাল হোসেন
- ◯ পলি খাতুন
- ◯ জিতেন্দ্র নাথ
- ◯ আমিরুল ইসলাম
- ◯ আবদুল হালিম
- ◯ শরিফুল হক

৫৩ গবেষণা সহকারির (১) মোবাইল নাম্বার

- ◯ 01787579854
- ◯ 01778599499
- ◯ 01711190152
- ◯ 01719538996
- ◯ 01300126112
- ◯ 01723253606
- ◯ 01992513568
- ◯ 01885386266
- ◯ 01712503535

৫৪ গবেষণা সহকারির (১) ই-মেইল এড্রেস

- ◯ sadiapreety97@gmail.com
- ◯ jiasminakter.tumpa@gmail.com
- ◯ jamaluddin1969@gmail.com
- ◯ hossainiqbal66@yahoo.com
- ◯ polyrayshuhi1984@gmail.com
- ◯ jitensdf1981@gmail.com
- ◯ amirulku33@gmail.com
- ◯ abdulhalimhamim577@gmail.com
- ◯ sharifulhaquedu@gmail.com

৫৫ গবেষণা সহকারির (২) নাম

- ◯ সাদিয়া প্রীতি
- ◯ জেসমিন আক্তার
- ◯ জামাল উদ্দিন মিয়া
- ◯ ইকবাল হোসেন
- ◯ পলি খাতুন
- ◯ জিতেন্দ্র নাথ
- ◯ আমিরুল ইসলাম
- ◯ শরিফুল হক
- ◯ আবদুল হালিম

৫৬ গবেষণা সহকারির (২) মোবাইল নাম্বার

- ◯ 01787579854
- ◯ 01778599499
- ◯ 01711190152
- ◯ 01719538996
- ◯ 01300126112
- ◯ 01723253606
- ◯ 01992513568
- ◯ 01885386266
- ◯ 01712503535

৫৭ গবেষণা সহকারির (২) ইমেইল এড্রেস

- ◯ sadiapreety97@gmail.com
- ◯ jiasminakter.tumpa@gmail.com
- ◯ jamaluddin1969@gmail.com
- ◯ hossainiqbal66@yahoo.com
- ◯ polyrayshuhi1984@gmail.com
- ◯ jitensdf1981@gmail.com
- ◯ amirulku33@gmail.com
- ◯ abdulhalimhamim577@gmail.com
- ◯ sharifulhaquedu@gmail.com
- ◯ jitensdf1981@gmail.comত
- ◯ abdulhalimhamim577@gmail.com

৫৮ গবেষণা সহকারির (৩) নাম

- ◯ আবদুল হালিম
- ◯ আমিরুল ইসলাম
- ◯ শরিফুল হক
- ◯ সাদিয়া প্রীতি
- ◯ পলি খাতুন
- ◯ জেসিমিন আক্তার
- ◯ জিতেন্দ্র নাথ
- ◯ ইকবাল হোসেন
- ◯ জামাল উদ্দিন মিয়া
- ◯ N/A

৫৯ গবেষণা সহকারির (৩) মোবাইল নাম্বার

- ◯ 01787579854
- ◯ 01778599499
- ◯ 01711190152
- ◯ 01719538996
- ◯ 01723253606
- ◯ 01992513568
- ◯ 01885386266
- ◯ 01712503535
- ◯ 01300126112
- ◯ N/A

৬০ গবেষণা সহকারির (৩) ইমেইল এড্রেস

- ◯ sadiapreety97@gmail.com
- ◯ jiasminakter.tumpa@gmail.com
- ◯ jamaluddin1969@gmail.com
- ◯ polyrayshuhi1984@gmail.com
- ◯ hossainiqbal66@yahoo.com
- ◯ amirulku33@gmail.com
- ◯ sharifulhaquedu@gmail.com
- ◯ N/A

ছবি সংযুক্তি
এফজিডি

Click here to upload file. (< 5MB)

ছবি সংযুক্তি
ডকুমেন্টস

Click here to upload file. (< 5MB)

Enter a date and time

yyyy-mm-dd hh:mm
