# Supplementary material for: Psychometric validation of a community healthcare scorecard used for marginalized populations in Bangladesh
Source: PLoS One. 2026 Aug 24;21(8):e0348720. doi: 10.1371/journal.pone.0348720 (PMC13502686; doi:10.1371/journal.pone.0348720)
Supplement: Supplementary file 2 [file pone.0348720.s002.pdf]

Community Scorecard (CSC) Questionnaire P37_Community Scorecard (CSC)_BRAC

Assessment of Public Services in Health, Education, and Social Security

2023

Introduction and Instructions

I am _ . I work at a research organization in Dhaka called CRD. On behalf of BRAC Bangladesh, CRD has undertaken a research study to assess various services provided to people from marginalized communities. As part of this study, we are conducting Community Score Cards and Focus Group Discussions (FGDs) with members of marginalized communities. Today, we would like to conduct a Community Score Card exercise and an FGD with you.

Duration of the discussion: 60–90 minutes. Participants should be selected from various marginalized communities. To ensure inclusivity and representation among respondents, diversity in age, educational background, occupation, and other relevant characteristics should be ensured.

Guidelines for Conducting FGDs / Community Score Card Sessions:

- Before starting the discussion, inform participants about the expected duration of the session.
- Please introduce yourself and begin the discussion on time.
- Please verify that the appropriate participants have been selected for the discussion.
- Please ensure that participants' signatures and relevant details are properly documented.
- Obtain consent from participants before taking notes and recording audio.
- Provide necessary examples and encouragement to help participants engage actively and willingly in the discussion.
- To verify immediate responses, ensure the participation of at least 2–3 participants.
- While providing examples, facilitating discussion, or arranging seating, consider local cultural norms and religious sensitivities.
- Please ensure that everyone has an equal opportunity to share their opinions or experiences.
- Before starting the discussion, assign a person to prevent interruptions or entry of unauthorized individuals during the session.

- Ensure that the seating arrangement complies with COVID-19 health and safety protocols (e.g., physical distancing, use of face masks by all participants, temperature checks, etc.).

Community Score Card (CSC): The Community Score Card (CSC) is a participatory, community-based monitoring and evaluation tool that helps ensure the assessment of the quality of public services such as health centers, schools, public transportation, water supply, and waste management from the citizen's perspective.

In social science research, various aspects such as human behavior, attitudes, characteristics, interests, intentions, and likelihood are measured. The Likert scale is a type of rating scale used for this purpose. In this study, the Likert rating scale will be used to develop the Community Score Card. Participant's responses will be collected through a structured questionnaire and ranked from the highest score (5) to the lowest score (1), or from positive (5) to negative (1), across different categories based on their ratings. This scoring helps to determine the level of importance or seriousness of an issue. Respondents must select one option on the scale for each question. These are always close-ended questions, meaning respondents will not have the opportunity to provide explanations.

Response options:

- 1 – Very Bad
- 2 – Bad
- 3 – Fair/Moderate
- 4 – Good
- 5 – Very Good

Section A: Background Information

No.	Question / Item	Options / Input
A1	Are you willing to participate in the Community Scorecard and FGD?	☐ Yes<br><br>☐ No
A2	Which division is the community located in?	☐ Dhaka<br>☐ Sylhet<br>☐ Chattogram<br>☐ Rajshahi<br>☐ Khulna<br>☐ Barishal<br>☐ Rangpur<br>☐ Mymensingh
A2(a)	Which district is the community located in? (Dhaka Division)	☐ Dhaka<br><br>☐ Faridpur<br>☐ Gazipur<br>☐ Gopalganj<br>☐ Kishoreganj<br>☐ Madaripur<br>☐ Manikganj<br>☐ Munshiganj<br>☐ Narayanganj<br>☐ Narsingdi<br>☐ Rajbari<br>☐ Shariatpur<br>☐ Tangail
A2(b)	Which district is the community located in? (Chattogram Division)	☐ Bandarban<br><br>☐ Brahmanbaria<br>☐ Chandpur<br>☐ Chattogram<br>☐ Cumilla<br>☐ Cox's Bazar<br>☐ Feni<br>☐ Khagrachhari<br>☐ Lakshmipur<br>☐ Noakhali<br>☐ Rangamati
A2(c)	Which district is the community located in? (Rajshahi Division)	☐ Bogura<br><br>☐ Joypurhat<br>☐ Naogaon<br>☐ Natore<br>☐ Chapainawabganj<br>☐ Pabna<br>☐ Rajshahi<br>☐ Sirajganj
A2(d)	Which district is the community located in? (Khulna Division)	☐ Bagerhat<br><br>☐ Chuadanga<br>☐ Jashore<br>☐ Jhenaidah<br>☐ Khulna<br>☐ Kushtia<br>☐ Magura<br>☐ Meherpur<br>☐ Narail<br>☐ Satkhira

No.	Question / Item	Options / Input
A2(e)	Which district is the community located in? (Barishal Division)	☐ Barguna<br>☐ Barishal<br>☐ Bhola<br>☐ Jhalokathi<br>☐ Patuakhali<br>☐ Pirojpur
A2(f)	Which district is the community located in? (Sylhet Division)	☐ Habiganj<br>☐ Moulvibazar<br>☐ Sunamganj<br>☐ Sylhet
A2(g)	Which district is the community located in? (Rangpur Division)	☐ Dinajpur<br>☐ Gaibandha<br>☐ Kurigram<br>☐ Lalmonirhat<br>☐ Nilphamari<br>☐ Panchagarh<br>☐ Rangpur<br>☐ Thakurgaon
A2(h)	Which district is the community located in? (Mymensingh Division)	☐ Jamalpur<br>☐ Mymensingh<br>☐ Netrokona<br>☐ Sherpur
A2(i)	Which Upazila is the community located in?	....
A2(j)	Which Union is the community located in?	....
A2(k)	Which Village is the community located in?	....
A2(l)	FGD Code (Division)	☐ Dhaka<br>☐ Sylhet<br>☐ Chattogram<br>☐ Rajshahi<br>☐ Khulna<br>☐ Barishal<br>☐ Rangpur<br>☐ Mymensingh
A2(m)	Code Number	....
A2(n)	Number of participants	....
A3	Age of Participant	☐ 18–40<br>☐ 41–60<br>☐ 60+
A4	Gender	☐ Female<br>☐ Male
A5	Which community do you belong to? (Select all that apply)	☐ Dalit<br>☐ Ethnic Minority / Indigenous<br>☐ Person with Disability<br>☐ Transgender / Third Gender<br>☐ Sex Worker<br>☐ Internally Displaced / Displaced<br>☐ Involved in Begging<br>☐ Elderly Person<br>☐ Urban Floating Population<br>☐ Bede (Nomadic Community)<br>☐ Hard-to-reach area (Char, Haor, Hill, Island)

No.	Question / Item	Options / Input
A6	Type of Residence	☐ Urban<br>☐ Rural
A7	Type of Religion	☐ Islam<br>☐ Hinduism<br>☐ Buddhism<br>☐ Christianity<br>☐ Others
A8	Occupation (Select all that apply)	☐ Agriculture / Fishery / Livestock<br>☐ Fisherman<br>☐ Cobbler<br>☐ Weaver / Handicraft<br>☐ Mason / Carpenter / Mechanic<br>☐ Day Laborer / Farm Laborer<br>☐ Rickshaw / Van Driver<br>☐ Transport Driver<br>☐ Garment Worker<br>☐ Sanitation Worker<br>☐ Small Business (< 10,000 Tk)<br>☐ Medium Business (10,000–1 Lakh Tk)<br>☐ Large Business (> 1 Lakh Tk)<br>☐ Government Job<br>☐ Private Job<br>☐ Homemaker<br>☐ Teacher<br>☐ Nurse<br>☐ Other Health Worker<br>☐ Public Representative<br>☐ Imam / Priest / Religious Leader<br>☐ Unemployed<br>☐ Student<br>☐ NGO Worker<br>☐ Sex Worker<br>☐ Begging<br>☐ Domestic Worker<br>☐ Other: ____________________

Section B: Government Healthcare Services

Please rate the following aspects of your nearest government hospital/health center.

**No.**	**Statement**	**1**	**2**	**3**	**4**	**5**
B10	There is a government hospital/health center near your area (it is easily accessible).	☐	☐	☐	☐	☐
B11	Services are available when you go to receive them.	☐	☐	☐	☐	☐
B12	Adequate health information is available (the information is understandable to you).	☐	☐	☐	☐	☐
B13	Equal treatment opportunities exist in government hospitals/health centers (you face no discrimination regarding medicine or seeing a doctor).	☐	☐	☐	☐	☐
B14	Services are provided in government hospitals without neglect (you are not subjected to bad behavior, hatred, or contempt).	☐	☐	☐	☐	☐
B15	There is a referral system in government hospitals/health centers (if they are unable to provide service, you are sent to the appropriate hospital through a proper process).	☐	☐	☐	☐	☐
B16	An institutional system for filing complaints exists in government hospitals (complaints can be made regarding the quality of service).	☐	☐	☐	☐	☐
B17	Proper action is taken when a complaint is made (remedial measures are taken).	☐	☐	☐	☐	☐
B18	There are enough doctors and nurses at the government hospital or health center nearest to you.	☐	☐	☐	☐	☐
B19	There are enough seats/beds, equipment, and medicine supplies at your nearest government hospital or health center.	☐	☐	☐	☐	☐
B20	Your nearest government hospital or health center has adequate drinking water, toilet facilities, etc.	☐	☐	☐	☐	☐
B21	Your nearest government hospital or health center has a breast-feeding corner.	☐	☐	☐	☐	☐
B22	Pregnant mothers in your neighborhood receive the government-mandated services they are entitled to (such as vaccinations, folic acid/vitamins, health workers visiting the mother, etc.).	☐	☐	☐	☐	☐
B23	Children in your neighborhood receive the government-mandated services they are entitled to (such as vaccinations, free healthcare up to 5 years of age, etc.).	☐	☐	☐	☐	☐

Scale: 1=Very Bad, 2=Bad, 3=Fair/Moderate, 4=Good, 5=Very Good

Section C: Government Primary Education

Please rate the following aspects of your local primary school.

No.	Statement	1	2	3	4	5
C24	The primary school is near your home/easy to commute to	☐	☐	☐	☐	☐
C25	At the school your child attends, teachers do not discriminate against your children in the classroom, playground, or exam hall	☐	☐	☐	☐	☐
C26	There is an opportunity to complain about the quality of education or related issues at the primary school	☐	☐	☐	☐	☐
C27	When a complaint is made, appropriate action is taken or redress is obtained	☐	☐	☐	☐	☐
C28	There are sufficient teachers at the primary school	☐	☐	☐	☐	☐
C29	The teaching by teachers at the primary school is understandable (boys-girls understand the class lessons)	☐	☐	☐	☐	☐
C30	Measures are taken to reduce the dropout rate of students at the primary school (teachers do home visits)	☐	☐	☐	☐	☐
C31	Post-COVID, there was sufficient initiative from teachers to bring students back to primary school	☐	☐	☐	☐	☐
C32	You, as a guardian, can participate in the parent-teacher meeting at the school	☐	☐	☐	☐	☐
C33	You are given the opportunity to speak at the parent-teacher meeting of the school	☐	☐	☐	☐	☐
C34	Is the education provided in the primary education institution of standard quality?	☐	☐	☐	☐	☐

Scale: 1=Very Bad, 2=Bad, 3=Fair/Moderate, 4=Good, 5=Very Good

Section D: Social Security Programs

Now please rate the following specific aspects:

No.	Statement	1	2	3	4	5
D35	If eligible for any social security program (e.g., old age allowance, food and cash assistance, maternity allowance, etc.), this service is easily available	☐	☐	☐	☐	☐
D36	Beneficiaries for these social security allowances/cards are selected by calling open meetings	☐	☐	☐	☐	☐
D37	Information about social security allowances/cards is available from local public representatives and government representatives	☐	☐	☐	☐	☐
D38	The information provided about social security allowances/cards is understandable	☐	☐	☐	☐	☐
D39	No discrimination is made (against you) in the selection of beneficiaries for providing social security allowances/cards	☐	☐	☐	☐	☐
D40	In the selection of beneficiaries (to receive allowances), nepotism, partisan favoritism, increasing vote banks, etc., are not done	☐	☐	☐	☐	☐
D41	No money (bribe or irregular payment) has to be given to receive social security allowances/cards	☐	☐	☐	☐	☐
D42	The amount of money, rice, etc., that is supposed to be given is provided in the correct amount	☐	☐	☐	☐	☐
D43	The materials (rice/wheat/oil) provided are of standard quality	☐	☐	☐	☐	☐
D44	If eligible but do not receive social security allowance/card or if materials are not standard, a complaint can be made	☐	☐	☐	☐	☐
D45	If a complaint is made, appropriate action is taken or redress is obtained	☐	☐	☐	☐	☐

Scale: 1=Very Bad, 2=Bad, 3=Fair/Moderate, 4=Good, 5=Very Good

Section E: Facilitating Team Details

Role	Name	Mobile Number	Email Address
Supervisor	Md. Anwarul Islam	01719268622	anowarul@crdbd.org
Enumerator 1	Md. Anowarul Islam	01719268622	anowarul@crdbd.org
Enumerator 2	Mahmuda Akter	01731088022	anna@crdbd.org
Enumerator 3	Tonmoy Nath	01873491169	tonmoy@crdbd.org
Enumerator 4	Sumaiya Akter	01885554108	sumaiya@crdbd.org
Enumerator 5	Md. Arafat Islam	01719027250	arafat@crdbd.org
Enumerator 6	Rahat Hannan	01521322486	rahat@crdbd.org
Enumerator 7	Nazia Nazneen	01687620675	nazia@crdbd.org
Research Assistant 1	Abdul Halim	01787579854	–
Research Assistant 2	Amirul Islam	01778599499	–
Research Assistant 3	Shariful Haque	01711190152	–
Research Assistant 4	Sadia Preeti	01719538996	sadiapreety97@gmail.com
Research Assistant 5	Poly Khatun	01723253606	polyrayshuhi1984@gmail.com
Research Assistant 6	Jesmin Akter	01992513568	jiasminakter.tumpa@gmail.com
Research Assistant 7	Jitendra Nath	01885386266	jitensdf1981@gmail.com
Research Assistant 8	Iqbal Hossain	01712503535	hossainiqbal66@yahoo.com
Research Assistant 9	Jamal Uddin Mia	01300126112	jamaluddin1969@gmail.com

Section F: Documentation

**FGD Documentation**
1. **FGD Summary:** ______ *(Click here to upload file, < 5MB)*
2. **FGD Photo:** ______ *(Click here to upload file, < 5MB)*
3. **Date and Time of FGD:** ______ (yyyy-mm-dd) ______ (hh:mm)

Thank you for your participation!
